# Growth differentiation factor-15 slows the growth of murine prostate cancer by stimulating tumor immunity

**DOI:** 10.1371/journal.pone.0233846

**Published:** 2020-06-05

**Authors:** Yasmin Husaini, Vicky Wang-Wei Tsai, Rakesh Manandhar, Hong Ping Zhang, Ka Ki Michelle Lee-Ng, Hélène Lebhar, Christopher P. Marquis, David A. Brown, Samuel N. Breit

**Affiliations:** 1 St Vincent’s Centre for Applied Medical Research, St Vincent’s Hospital and University of New South Wales, Sydney, New South Wales, Australia; 2 School of Biotechnology and Biomolecular Sciences, University of New South Wales, Sydney, New South Wales, Australia; 3 The Institute for Pathology and Clinical Research, New South Wales Health Pathology and The Westmead Institute for Medical Research, Westmead Hospital and University of Sydney, Wesmead, New South Wales, Australia; University of Minnesota Twin Cities, UNITED STATES

## Abstract

Growth Differentiation Factor-15 (GDF15) is a divergent TGF-beta superfamily cytokine that is overexpressed by most cancers and is induced by anticancer therapy. Transgenic and induced animal models suggest that it protects from cancer development but the mechanisms are uncertain. We investigated the role of immunity in GDF15 induced reduction in prostate cancer (PCa) growth. The C57BL/6 transgenic TRAMP prostate cancer prone mice were bred with mice that were immunodeficient and/or systemically overexpressed GDF15. We developed a novel orthotopic TRAMP PCa model in which primary TRAMP tumor cells were implanted into prostates of mice to reduce the study time. These mice were administered recombinant mouse GDF15, antibody to CD8, PD1 or their respective controls. We found that GDF15 induced protection from tumor growth was reversed by lack of adaptive immunity. Flow cytometric evaluation of lymphocytes within these orthotopic tumors showed that GDF15 overexpression was associated with increased CD8 T cell numbers and an increased number and proportion of recently activated CD8^+^CD11c^+^ T cells and a reduced proportion of "exhausted" CD8^+^PD1^+^ T cells. Further, depletion of CD8 T cells in tumor bearing mice abolished the GDF15 induced protection from tumor growth. Infusion of GDF15 into mice bearing orthotopic TRAMP tumor, substantially reduced tumor growth that was further reduced by concurrent PD1 antibody administration. GDF15 overexpression or recombinant protein protects from TRAMP tumor growth by modulating CD8 T cell mediated antitumor immunity and augments the positive effects of anti-PD1 blockers.

## Introduction

Growth Differentiation Factor-15 (GDF15) is a divergent TGF-beta superfamily cytokine [1], most closely linked to the glial derived neurotrophic factor family because its recently identified receptor Glial-derived neurotrophic factor receptor **α**-like (GFRAL) is an orphan member of the Glial-derived neurotrophic factor receptor **α** (GFRa) family [2–5]. GFRAL and other members of the GFR**α** family signal through the Ret tyrosine kinase co-receptor, which can propagate signals through many different pathways. GDF15 does not utilize the classical Transforming growth factor-b receptor I (TBRI) and II (TBRII) and does not signal through the canonical smad signaling pathway family [2, 3, 5].

GDF15 is a stress response cytokine whose expression and serum levels may increase with injury, inflammation and malignancy and whose biology has been recently extensively reviewed elsewhere [6]. In normal humans, GDF15 circulates at a concentration of about 200–1200 pg/ml but these levels rise in some diseases. GDF15 is overexpressed by the majority of cancers and its serum levels rise broadly in line with stage and extent of disease [6]. Expression can also be increased by anti-cancer therapies such as radiotherapy and chemotherapy [7–11]. Especially in advanced cancers, serum levels can rise markedly by up to 10–100 fold leading to anorexia and loss of lean and fat mass and the development of an anorexia/cachexia syndrome [12, 13]. This is mediated largely through systemic GDF15 induced modulation of central appetite regulatory circuits [12, 14].

There is considerable epidemiological data linking GDF15 to cancers [6]. A polymorphism (H6D) in the *GDF15* gene manifests as a non-conservative amino acid change in the sequence of the mature domain of GDF15 and alters the risk and behavior of colorectal and prostate cancers [15–18]. Serum levels of GDF15 progressively rise with the evolution of colonic polyps to colorectal cancer [16]. These increased levels predict a worse outcome in patients with colorectal carcinoma [16, 19] and many other cancers [6]. However, these may not always be a good reflection of local availability of GDF15 within the tumor. Especially in cancer, GDF15 is sometimes secreted in an unprocessed form with its propeptide still attached [20, 21]. As the propeptide contains a heparan sulphate binding motif, this form of GDF15 can bind to extracellular matrix and thus remain localized to the tumor [20], from where it might be slowly released to provide a local pool of GDF15. This tumor localized GDF15 may be important because prostate tumors from patients with early cancer and with increased staining for tumor associated GDF15 have a significantly better prognosis [20].

The role of GDF15 in the biology of cancer has been studied using a number of different approaches, which have yielded somewhat contradictory results. In vitro studies are difficult to interpret as at least one major commercial supplier of GDF15 has sold a product known to be contaminated by TGF-beta and this is the likely cause of studies erroneously demonstrating GDF15 induced smad signaling [22–24]. In vivo studies using transfected tumor cell lines which are xenografted into immunodeficient mice have suggested, overall, that GDF15 may facilitate tumor growth and spread [6].

Transgenic and induced cancer models more closely reflect the progressive molecular changes of carcinogenesis, more often mimic early cancer and use rodents with intact immune systems. GDF15 overexpressing mice are protected from urethane induced lung cancer [25] and azoxymethane induced colonic cancers [26]. Additionally, utilizing the mutant adenomatous polyposis coli (APC) gene mouse model of colonic polyps and cancer, mice overexpressing GDF15 are protected from the development of polyps and cancer [26]. *Apc* mutant mice loose NSAID induced protection from the development of colonic polyps if they are germline *Gdf15* gene deleted [27]. These findings in mice with colonic polyps might translate to humans is suggested by data that human serum GDF15 levels are directly influenced by the presence of adenomatous polyps and elevated GDF15 serum levels rapidly reduce with removal of the polyp [28]. Further, only patients that display a rise in serum GDF15 levels with NSAID use are protected from adenomatous polyp development [29].

We have been studying the role of GDF15 in the spontaneous development of prostate cancer (PCa) in C57BL/6 background TRAMP mice in which we have genetically manipulated GDF15 expression. We have used C57BL/6 background mice, as unlike FVB background mice, they do not develop a high proportion of neuroendocrine PCa, which is rare in humans [30,31]. Further, unlike many cancers, TRAMP PCa do not express GDF15 [32]. Thus, this model is most reflective of the substantial minority of prostate cancer patients that do not express GDF15. These studies indicate that TRAMP mice with a germline deletion in *Gdf15* develop PCa more quickly, have larger tumors and die earlier than TRAMP mice with wild type *Gdf15* [33]. On the other hand, TRAMP mice with transgenic overexpression of GDF15 develop PCa much more slowly, have lower histological grade, smaller tumors and live much longer than either TRAMP mice with WT *Gdf15* [32]. Such a protective role is supported by studies indicating that tumor tissue localized GDF15 staining is associated with a better outcome in patients with early stage PCa [20]. Interestingly however, with aging TRAMP mice overexpressing GDF15 develop more metastases than the other genotype TRAMP mice, suggesting that GDF15 may play a dual role in cancer [32]. It may protect from the development, growth and spread of early cancer but with advanced disease facilitate cancer spread. This dual role in cancer is seen with other cytokines, most prominently TGF-beta. Whatever the relationship of GDF15 to cancer outcome, because of its widespread expression by cancers and induction by many cancer therapies, understanding the role of GDF15 in cancer is likely to be of substantial clinical relevance as any impact on its expression is likely to have clinical consequences outside of GDF15’s role in cancer anorexia/cachexia syndrome.

One potential mechanism that may explain the differences in the action of GDF15 on early and advanced cancer and the differences in the data from induced or transgenic cancer models and mouse tumor xenograft models is modulation of antitumor immunity. To investigate this, we have again utilized the C57BL/6/TRAMP model of spontaneous PCa in which we have both studied and manipulated the immune environment.

## Materials and methods

### Ethical approval of the study

All animal experiments were approved by the Garvan/St. Vincent’s Hospital Animal Ethics Committee (Ethic approval number: 13/08 and 16/05). The experiments were performed in accordance with guidelines of Australian Code for the Care and Use of Animals for Scientific Purposes (8^th^ Ed).

### Mice

Transgenic PCa prone TRAMP mice [34] and mice overexpressing GDF15 under the control of the murine CSF-1 (fms) promoter (TRAMP^fmsmic-1^) have been previously described [12, 32, 35]. TRAMP^rag-/-^ mice that lacked adaptive immunity were generated by crossing TRAMP^+/-^ females with homozygous male WT^rag-/-^ (B6.129S7-Rag1^tm1Mom^/J, The Jackson Laboratory) on a C57BL/6 background. A double transgenic MIC-1^fms/rag-/-^ mouse line was generated by crossing homozygous MIC-1^fms^ females with homozygous WT^rag-/-^ males. Triple transgenic TRAMP^fmsmic/rag1-/-^ mice were produced by crossing TRAMP^rag-/-^ females with MIC-1^fms/rag-/-^ males. All mice were on a C57BL/6 background. For mouse genotyping see S1 Data.

### Survival study

TRAMP, TRAMP^fmsmic-1^, TRAMP^rag-/-^ and TRAMP^fmsmic/rag1-/-^ mice (n = 35/group) at 4–6 weeks of age were assigned to a survival study. Mice were housed in a pathogen-free animal facility at 22–23 °C with a 12:12 h light-dark cycle. Survival study was run for duration of 18 months. Mice were weighed weekly and checked twice a week for the presence of tumor by palpating of their abdomen. A 5% of morbidity and mortality was approved by ethics committee in the study but mice were euthanized on the same day as they reached ethical endpoint for the tumor size (11 mm x 11 mm) or meet any other ethical endpoint criteria such as: if the animal exhibits a ≥ 20% body weight loss, hunched posture, ruffled hair coat, a Body Condition Score of ≤ 2, bleeding from any orifices and has impaired mobility that restricts feeding, drinking and normal behaviors. Out of 35 mice per group, 3 mice in TRAMP group, 4 mice in TRAMP^fmsmic-1^ group, 3 mice in TRAMP^rag-/-^ group and 5 mice in TRAMP^fmsmic/rag1-/-^ group were euthanized for the reasons other than tumor growth. All the mice that reached ethical endpoint were killed by trained staff (Biological Testing Facility, Garvan Institute of Medical Research) by first anaesthetizing mice using Isoflurane (3% at a flow rate of 3 litres of oxygen per minute) and then by cervical dislocation. Mice killed for a reason other than seminal vesicle (SV) or prostate tumor were excluded from the study. Survival distribution was estimated by Kaplan-Meier method as previously described [32].

### Primary tumor size in transgenic mice

Prostate tumor growth was compared in a group of TRAMP (n = 22), TRAMP^fmsmic-1^ (n = 15), TRAMP^rag-/-^ (n = 18) and TRAMP^fmsmic/rag1-/-^ (n = 14) mice at 25 weeks age. At necropsy, prostate tumor was excised from the GU complex and tumor weight was recorded. Prostate weight was normalized for the mouse body weight (tumor wt/body wt).

### Orthotopic TRAMP tumor transplantation into WT, MIC-1^fms^, Rag^-/-^ and MIC-1^fms/rag-/-^ mice

To reduce the study time of 30–50 weeks in TRAMP mice, a period that is markedly increased by breeding of double or triple transgenic mice, we developed a more efficient approach based on tumor engraftment by intraprostatic injection of primary tumor cells from a donor TRAMP mouse into up to 50–60 recipient mice. We have called this the **O**rthotopic **T**RAMP **T**umor **E**ngraftment **M**odel (OTTEM). A primary prostate tumor from a TRAMP mouse was excised, weighed, cut into small pieces of about 3 mm^3^ and then teased through a 100 u strainer. The cells were then washed and passed through 70 u and 40 u strainers to generate a single cell suspension. The tumor and dispersed cells were kept in DMEM with 10% FBS and maintained on ice throughout the tumor processing. Cells were given a final wash in serum free DMEM and the viable tumor cells were counted and the number adjusted to 10^6^ cells/30 ul. Following surgical exposure and bladder retraction, a 30 ul volume of tumor cells was injected using a 0.5 ml insulin syringe by direct vision, into the dorsal prostate lobe where spontaneous TRAMP cancers develop. By ~4–6 weeks the TRAMP tumor cells orthotopically engrafted into WT syngeneic C57BL/6J mice, resulted in a tumor of about 1 cm diameter in the majority of mice. For experiments, donor tumors from TRAMP, TRAMP^rag-/-^ or TRAMP^MIC-/-^ mice were engrafted into a number of different WT or transgenic C57BL/6J background mice. Prostate tumor were excised and weighed at the end of the experiment, but in this instance not normalized to body weight. Since a different primary donor tumor is used for each experiment, there is experiment to experiment variability in the engrafted tumor growth, based on the aggressiveness of the donor tumor.

### Systemic delivery of recombinant GDF15 by mini-osmotic pump

The in-house production and purification of recombinant mouse GDF15 (muGDF15), has been previously described [36]. One million primary prostate tumor cells from a donor TRAMP mouse were implanted into WT (n = 20) mouse prostates by intraprostatic injections as described above. On the same day 10 mice were implanted subcutaneously with a 28-day mini-osmotic pump (Model 2004, ALZET Osmotic pump, Cupertino, CA) containing recombinant muGDF15 to deliver 0.5 ug GDF15/g BW/day or vehicle, as previously described [37]. After 28 days, mice were sacrificed and prostate tumors were excised and weighed.

### Systemic recombinant GDF15 and anti-PD1 antibody treatment

One million primary prostate tumor cells from a donor TRAMP mouse were implanted into prostates of 48 mice by intraprostatic injections, using the OTTEM model described above. After 72 hours post tumor implantation, 24 mice were implanted subcutaneously with 28-day mini-osmotic pump filled with recombinant muGDF15 to deliver 0.5 ug GDF15/g BW/day. The other 24 mice were implanted with pump containing vehicle. Starting from the day of osmotic pump implantation, half of both groups were also given twice weekly intraperitoneal injections of 250 ug/mouse rat anti-mouse PD1 monoclonal antibody (Clone RMP1-14, Bio X Cell) and the rest received 250 ug/mouse isotype control antibody (Rat IgG2a, Clone 2A3, Bio X Cell). After 28 days mice were sacrificed and prostate tumors were excised and weighed.

### Detection of TRAMP tumor infiltrating lymphocytes

One million dispersed cancer cells from a primary TRAMP^MIC-/-^ prostate tumor were injected into WT (n = 15) and MIC-1^fms^ (n = 15) mouse prostate using the OTTEM model described above. At 5–6 weeks, intraprostatic tumors were excised and weighed. Tumors were cut into small pieces of about 3 mm^3^ and then teased through a 100 u strainer. Tumor cells were then washed with staining wash buffer (SWB, PBS with 2% FBS and 1 mM EDTA), passed through 70 u and 40 u strainers to generate a single cell suspension. Cell numbers were counted and one million cells from each tumor were centrifuged and blocked with purified anti-mouse CD16/CD32 monoclonal antibody clone 2.4G2 (0.5 ug/million cells in 50 ul) on ice for 10 minutes. Cells were then stained with the fluorochrome labelled anti mouse monoclonal antibodies (see S1 Table), for 20–30 minutes on ice in the dark. Stained cells were washed twice with SWB and subjected to multiparameter flow cytometry on LSRFortessa^™^ X-20 flow cytometer with BD FACSDiva^™^ software. Flow cytometry data was analyzed using FlowJo following the gating strategy shown in S1 Fig and described in S1 Data.

In order to compare proportion of tumor infiltrating CD8 T cells expressing PD1, dispersed tumor cells were stained with fluorochrome labeled anti mouse monoclonal antibody to identify, leukocytes (CD45^+^), CD4 T cells (CD3^+^CD4^+^) CD8 T cells (CD3^+^CD8^+^) and PD1 expressing CD8T cells (CD3^+^CD8^+^PD1^+^) (see panel 2, S1 Table). Gating strategy for the CD3^+^CD8^+^PD1^+^ cells is shown in S2 Fig.

### *In vivo* CD8 T cell depletion of tumor bearing mice

We have depleted CD8 T cells in mice as previously described [38]. We validated the procedure first as described in S1 Data and S2 Table. We next depleted CD8 T cells from WT (n = 6) and MIC-1^fms^ (n = 5) mice by injecting 400 ug of rat anti-CD8-α monoclonal antibody one day prior to intraprostatic tumor implantation. A control group of WT (n = 6) and MIC-1^fms^ (n = 5) mice received 400 ug of isotype matched control antibody. On the next day we injected, TRAMP^MIC-/-^ primary prostate tumor cells as mentioned above into prostate of all the above mice. CD8 depletion of mice was maintained by injecting same dose of anti CD8-α or control antibody, as above, twice weekly. After 5 weeks, mice were sacrificed; prostate tumors excised and weights were recorded.

### Statistical analysis

Statistical evaluations of all the experiments were performed with GraphPad Prism software version 7 (GraphPad Software, San Diego, CA, USA). All the data are presented as the mean ± standard error of the mean (s.e.m.). Comparisons between groups were made using unpaired *t* tests. Survival curves were analyzed by Kaplan–Meier analysis and log-rank statistic is reported. A *p* value of 0.05 or less was considered statistically significant.

## Results

### GDF15 associated increase in survival of TRAMP mice is reversed in the absence of adaptive immunity

**Fig 1:**
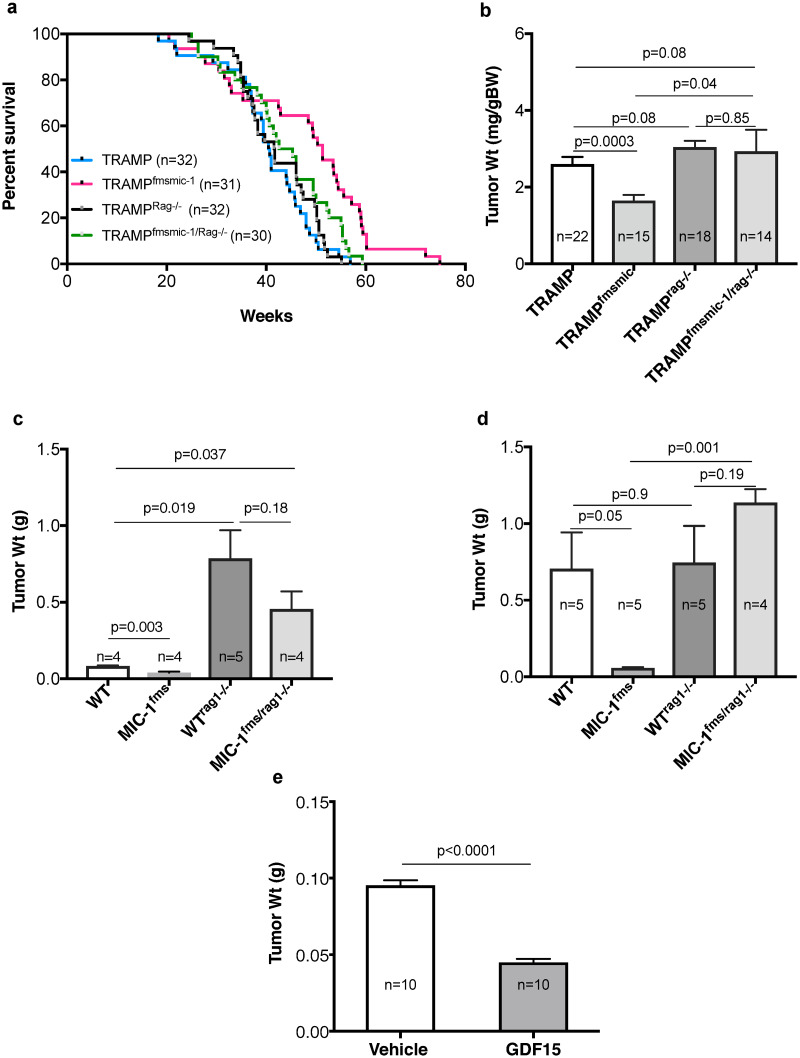
Effect of immunity on GDF15 mediated protection from TRAMP tumor growth. (a) Comparison of survival curves for C57BL/6 background TRAMP mice, TRAMP^fmsmic-1^ mice that also overexpress GDF15, immunodeficient TRAMP^rag-/-^ mice and TRAMP^fmsmic/rag1-/-^ mice that both overexpress GDF15 and lack adaptive immunity. Overall survival of individual mice from birth to death was plotted using the Kaplan-Meier method. (b) Body weight corrected prostate tumor weights of TRAMP, TRAMP^fmsmic-1^, TRAMP^rag-/-^ and TRAMP^fmsmic/rag1-/-^ mice at 25 weeks are presented as mean ± s.e.m. (c) Prostate tumor weights of WT, MIC-1^fms^, WT^rag-/-^ and MIC-1^fms/rag-/-^ mice 5 weeks after orthotopic implantation of primary tumor cells from a TRAMP mouse lacking adaptive immunity because of a germline deletion of the *Rag-1* gene (TRAMP^rag-/-^). Results are presented as mean tumor weight ± s.e.m. (d) Prostate tumor weight of C57BL/6 mice that are WT, overexpress GDF15 (MIC-1^fms^), lack adaptive immunity (WT^rag-/-^) and or both overexpress GDF15 and lack adaptive immunity (MIC-1^fms/rag-/-^) 5 weeks after orthotopic engraftment of prostate tumor cells from a single C57BL/6 TRAMP mouse with a germline deletion of *Gdf15* (TRAMP^MIC-/-^). The results are presented as mean tumor weight ± s.e.m. (e) To evaluate the effect of exogenous recombinant muGDF15 on tumor growth, C57BL/6 WT mice were implanted orthotopically with primary TRAMP^MIC-/-^ prostate tumor cell and infused with vehicle or muGDF15 using 28-day mini-osmotic pump. Mice were sacrificed at 28 days. Results are presented as mean tumor weight ± s.e.m. All tumor weight data was analyzed using an unpaired 2-tailed t test and the *p* values are shown on the graphs.

GDF15 reproducibly increases survival and reduces cancer growth of PCa prone transgenic TRAMP mice [32, 33]. In order to determine if this is mediated by adaptive immunity, we compared overall survival of C57BL/6J background TRAMP mice and TRAMP mice that overexpress GDF15 (TRAMP^fmsmic-1^) with their counterparts that also lack an adaptive immune system because of a concurrent deletion of the *Rag1* gene (TRAMP^Rag1-/-^, TRAMP^fmsmic/rag1-/-^; Fig 1a). We monitored this cohort of TRAMP, TRAMP^fmsmic-1^, TRAMP^Rag1-/-^ and TRAMP^fmsmic/rag1-/-^ mice till they reached ethical endpoint. Kaplan-Meier survival analysis again demonstrated, as we have previously reported, that TRAMP^fmsmic-1^ mice have a significantly longer median survival of 51.3 weeks compared to 40.6 (Fig 1a; *p* = 0002) in TRAMP mice. Mice lacking overexpression of GDF15, with or without adaptive immunity, TRAMP, TRAMP^rag-/-^, respectively had a similar overall survival (median survival 40.6 weeks and 41.6 weeks respectively, *p* = 0.5, log-rank test). However, the longer survival of TRAMP^fmsmic-1^ was abrogated in the triple transgenic TRAMP^fmsmic/rag1-/-^ mice (median survival 51.3 and 43.9 weeks respectively, *p* = 0.02, log-rank test). The absence of adaptive immunity reduced the median survival of TRAMP^fmsmic/rag1-/-^ mice such that it was similar to that of TRAMP mice (median survival 43.9 and 40.6 weeks respectively, *p* = 0.08, log-rank test). These data indicate that the improvement in survival of GDF15 overexpressing TRAMP^fmsmic-1^ mice requires intact adaptive immunity.

### GDF15 associated protection from TRAMP tumor growth is reversed in the absence of adaptive immunity

To directly assess the impact of lack of adaptive immunity on our previously observed GDF15 mediated reduction in PCa growth, we compared prostate tumor growth in a cohort of TRAMP (n = 22), TRAMP^fmsmic-1^ (n = 15), TRAMP^rag-/-^ (n = 18) and TRAMP^fmsmic/rag1-/-^ (n = 14) mice (Fig 1b). Mice were sacrificed at 25 weeks of age and the prostate tumors were isolated and weighed. There was 36.8% reduction in normalized tumor weight in TRAMP^fmsmic-1^ mice (Fig 1b, *p* = 0.0003), consistent with our previous findings [32]. There was no significant difference in the normalized prostate tumor weight between TRAMP and TRAMP^rag-/-^ or TRAMP^fmsmic/rag1-/-^ mice (Fig 1b, *p* = 0.08). This also demonstrated that the tumor size reduction that was present in the TRAMP^fmsmic-1^ cohort was abolished in the TRAMP^fmsmic/rag1-/-^ group that overexpressed GDF15 but also lacked adaptive immunity (Fig 1b). Consistent with the survival study above, these data indicate that protection from prostate tumor growth in GDF15 overexpressing TRAMP mice requires intact adaptive immunity.

### The immune system plays an important role in control of growth of orthotopically transplanted TRAMP prostate tumors

To be able to more efficiently study the impact of lack of adaptive immunity on GDF15 mediated reduction in PCa growth, we developed and used **O**rthotopic **T**RAMP **T**umor **E**ngraftment **M**odel. We isolated a TRAMP tumor from a donor mouse then orthotopically implanted cells from this tumor into the prostates of multiple syngeneic genetic background recipient experimental mice. We implanted primary tumor cells isolated from a single TRAMP^rag-/-^ prostate tumor into 4–5 per group of each of the following C57BL/6J background mice, WT, MIC-1^fms^, WT^rag-/-^ and MIC-1^fms/rag-/-^ mice. After 5wks, mice were sacrificed and their prostate tumors isolated and weighed (Fig 1c). TRAMP^rag-/-^ tumor cells engrafted into WT mice show marked reduction in tumor growth compared to when the same tumor cells are engrafted into WT^rag-/-^ (Fig 1c). This growth is further reduced when TRAMP^rag-/-^ cells are engrafted into GDF15 overexpressing transgenic MIC-1^fms^ mice (Fig 1c). In fact the weights of engrafted prostates excised from the MIC-1^fms^ mice are not significantly different from those of age matched normal MIC-1^fms^ mice (0.0396±0.007g and 0.037±0.001g respectively, *p* = 0.75) suggesting that all tumor cells might well have been eliminated in this group. The size of tumors in MIC-1^fms/rag-/-^ mice, that overexpressed GDF15 but lacked adaptive immunity, engrafted with TRAMP^rag-/-^ tumor cells, did not differ from that of the same tumor cells engrafted into WT^rag-/-^. The cells from the TRAMP^rag-/-^ prostate tumor have never come in to contact with an adaptive immune system and will thus displayed increased sensitivity to adaptive immune mediated killing. For this reason, whilst adaptive immunity has no visible impact on the growth of tumors growing spontaneously in TRAMP mice (Fig 1b) it has a major impact on TRAMP^rag-/-^ tumor cells (Fig 1c) and that impact is even greater in GDF15 overexpressing transgenic MIC-1^fms^ mice. However, GDF15 cannot exert its protection from tumor growth without intact adaptive immunity.

### GDF15 protection from growth of orthotopically transplanted prostate tumor in GDF15 overexpressing mice is reversed in Rag^-/-^ mice

We next implanted isolated TRAMP primary prostate tumor cells into 5 per group of each of the following C57BL/6J background mice, WT, MIC-1^fms^, WT^rag-/-^ and MIC-1^fms/rag-/-^. After 5wks, mice were sacrificed and their prostate tumors isolated and weighed. The pattern of tumor growth in these mice (Fig 1d) was similar to that in double and triple transgenic mice reported above (Fig 1b). GDF15 overexpressing MIC-1^fms^ mice again had significantly smaller prostate tumors than WT mice (Fig 1d, *p* = 0.05). There was no significant difference in the prostate tumor weight between WT and WT^rag-/-^ mice (Fig 1d, *p* = 0.9) nor was there any difference in tumor weight between WT^rag-/-^ and MIC-1^fms/rag-/-^ groups (Fig 1d, *p* = 0.19). Again, the protection from tumor growth in the MIC-1^fms^ group was reversed when these mice were also immunodeficient because of concurrent *Rag1* gene deletions (Fig 1d). Like the data from transgenic TRAMP mice, the orthotopic tumor size data suggests that the GDF15 mediated protection from growth of engrafted TRAMP tumors requires intact adaptive immunity.

### Systemic recombinant GDF15 protects from PCa development

To determine if systemic GDF15 would also protect mice from the growth of TRAMP PCa, we first implanted the prostates of 20 WT mice with tumor cells from TRAMP with a normal immune system and bearing no other genetic modifications, using the OTTEM model. On the same day as tumor implantation, using a 28-day mini-osmotic pump, we started infusion of recombinant muGDF15 (0.5 ug/g BW/day) or vehicle for 28 days. Serum GDF15 levels in the cytokine infused group raises to about 6–8 ng/ml, which is less than the 10–14 ng/ml usually found in the blood of MIC-1^fms^ mice. muGDF15 treated mice had markedly reduced prostate size compared to vehicle treated mice (Fig 1e, *p*<0001). Thus in the modest doses used, systemic GDF15 provides substantial protection from early PCa growth, indicating its effects on adaptive immunity can be reproduced with systemic delivery of this protein.

### GDF15 protection from TRAMP prostate cancer growth is reversed by anti-CD8 antibody

**Fig 2:**
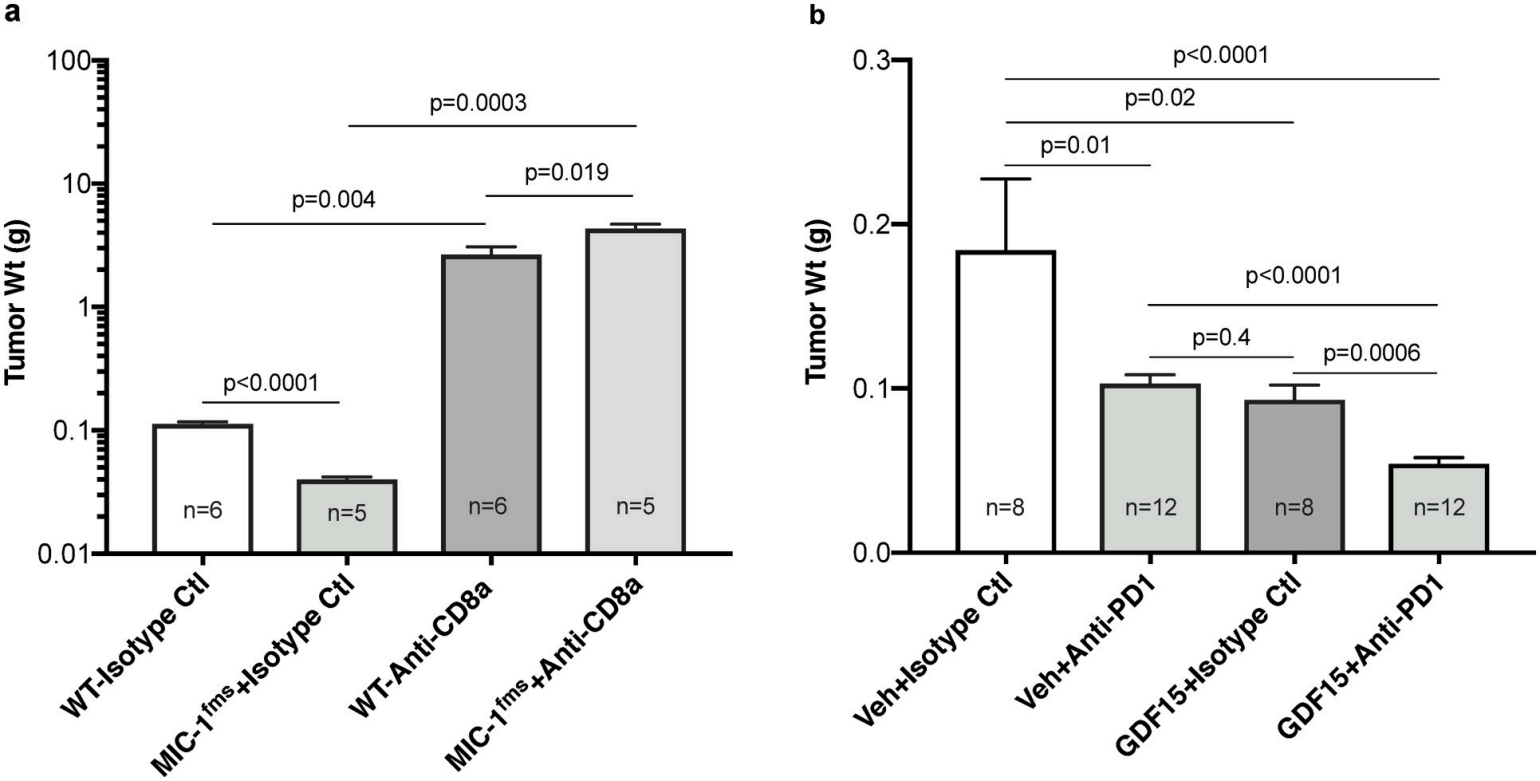
Modulation of GDF15 mediated protection from TRAMP tumor growth. (a) All mice were injected twice weekly with anti-CD8-α monoclonal antibody or isotype matched anti KLH antibody starting one day before tumor cell implantation. TRAMP^MIC-/-^ primary prostate tumor cells were engrafted orthotopically into WT and MIC-1^fms^ mice and 35 days later mice were sacrificed and the tumors dissected and weighed. Results are presented as mean tumor weight ± s.e.m. (b) WT mice were engrafted orthotopically with TRAMP^MIC-/-^ tumor cells. Three days later mice were infused with recombinant muGDF15 (0.5 ug/g BW/day) or vehicle via 28-day mini-osmotic pump and also given twice-weekly intraperitoneal injections of anti-PD1 monoclonal antibody (250 ug/mouse) or isotype control antibody. After 28 days mice were sacrificed and prostate tumors were excised and weighed. The graph represents compiled data from two experiments. Results are presented as mean tumor weight ± s.e.m. All tumor weight data was analyzed using unpaired 2-tailed t test and the *p* value are shown on the graphs.

A major cell responsible for adaptive immune mediated tumor killing is the CD8 T cell. To test if the protection from TRAMP PCa growth was mediated by this cell, we orthotopically implanted TRAMP^MIC-/-^ tumor from one mouse into groups of WT and MIC-1^fms^ mice. One day before implantation, we started injecting 400 ug of anti-CD8-α monoclonal antibody or 400 ug of Isotype matched anti KLH monoclonal antibody, intraperitoneally twice weekly for 35 days at which time mice were sacrificed and the tumors dissected and weighed. At necropsy, isotype control antibody treated MIC-1^fms^ mice had significantly smaller tumor than isotype control antibody treated WT mice (Fig 2a, *p*<0.0001), consistent with the previously reported actions of GDF15. After CD8 T cell depletion WT and MIC-1^fms^ tumor grew larger than their respective isotype control antibody treated mice (Fig 2a). However, CD8 T cell depleted MIC-1^fms^ mice had significantly larger tumors than CD8 T cell depleted WT mice (Fig 2a, *p* = 0.019). Whilst some other immune cells such as dendritic cells may express CD8-α, in the context of the other available data, this suggests that CD8 T cells mediate the GDF15 induced reduction in TRAMP tumor growth and development.

### Systemic recombinant GDF15 provides additional protection from PCa development to that provided by anti-PD1

Our data suggests that systemically delivered muGDF15 protects from TRAMP tumor growth by stimulating adaptive immunity. We therefore wanted to test whether GDF15 would provide additional protection to that from antibody to PD1, a well-characterized stimulator of antitumor immunity, being used in the clinic. Forty-eight WT mice in two batches were implanted intraprostatically with PCa cells derived from a single TRAMP^MIC-/-^ donor mouse using the OTTEM model. On this occasion, to ensure that GDF15 was not acting by altering primary tumor cell implantation, the commencement of treatment was delayed for 3 days after which muGDF15 (0.5 ug/g BW/day) or vehicle via mini-osmotic pump was commenced. Mice were then given twice-weekly intraperitoneal injections of anti-mouse PD1 monoclonal antibody (250 ug/mouse) or isotype control antibody. After 28 days mice were sacrificed and prostate tumors were excised and weighed. Treatment with vehicle plus anti-PD1 or muGDF15 plus isotype control antibody displayed reduced prostate sizes compared to their respective control mice (Fig 2b, *p* = 0.01 and *p* = 0.02 respectively). Treatment of mice with both anti-PD1 antibody and muGDF15 further substantially reduced tumor growth over either of the two treatments alone (Fig 2b, *p*<0.0001 and *p* = 0.0006 respectively). In the combined treatment group, the average prostate weight of 0.054+/-0.003 g was indistinguishable from a group of 5 age matched WT mice prostate of 0.050 g+/-0.001 g (*p* = 0.346), suggesting that the tumor may have been almost, or completely eliminated. Thus, addition of muGDF15, to a well characterized stimulator of tumor immunity results in significant additional benefit.

### GDF15 alters the tumor infiltrating lymphocytes of TRAMP prostate tumors

Our earlier data suggested that GDF15 mediated protection from tumor growth required intact adaptive immunity and CD8 T cells. To further investigate adaptive immunity, we studied the lymphocytic infiltrate of TRAMP tumors. In order to determine whether GDF15 overexpression altered the lymphocyte composition of tumors, using the OTTEM model, we orthotopically engrafted both WT and MIC-1^fms^ mice with cells derived from prostate tumors of TRAMP^MIC-/-^ mice. At 5–6 weeks, implanted intra-prostatic tumors from WT and MIC-1^fms^ mice were excised and weighed an average of 3.0 g and 1.5 g respectively (*p* = 0.0001). Based on weight, a known proportion of the tumor from each mouse was then processed into a single cell suspension. Without any purification or fractionation, these cells were stained with fluorochrome labeled monoclonal antibodies and one million cells were subjected to multiparameter flow cytometry on BD LSRFortessa^™^ X-20 flow cytometer, followed by data analysis using FlowJo. Panels were designed with monoclonal antibodies to: *Panel 1*: CD45, CD3, CD4, CD8, NK1.1, CD11c, CD11b, and B220. Using the gating strategy shown in S1 Fig, focusing on the CD45 positive population, these antibodies allowed us to identify major T cell subsets (CD4, CD8), NK cells and B cells.

**Fig 3:**
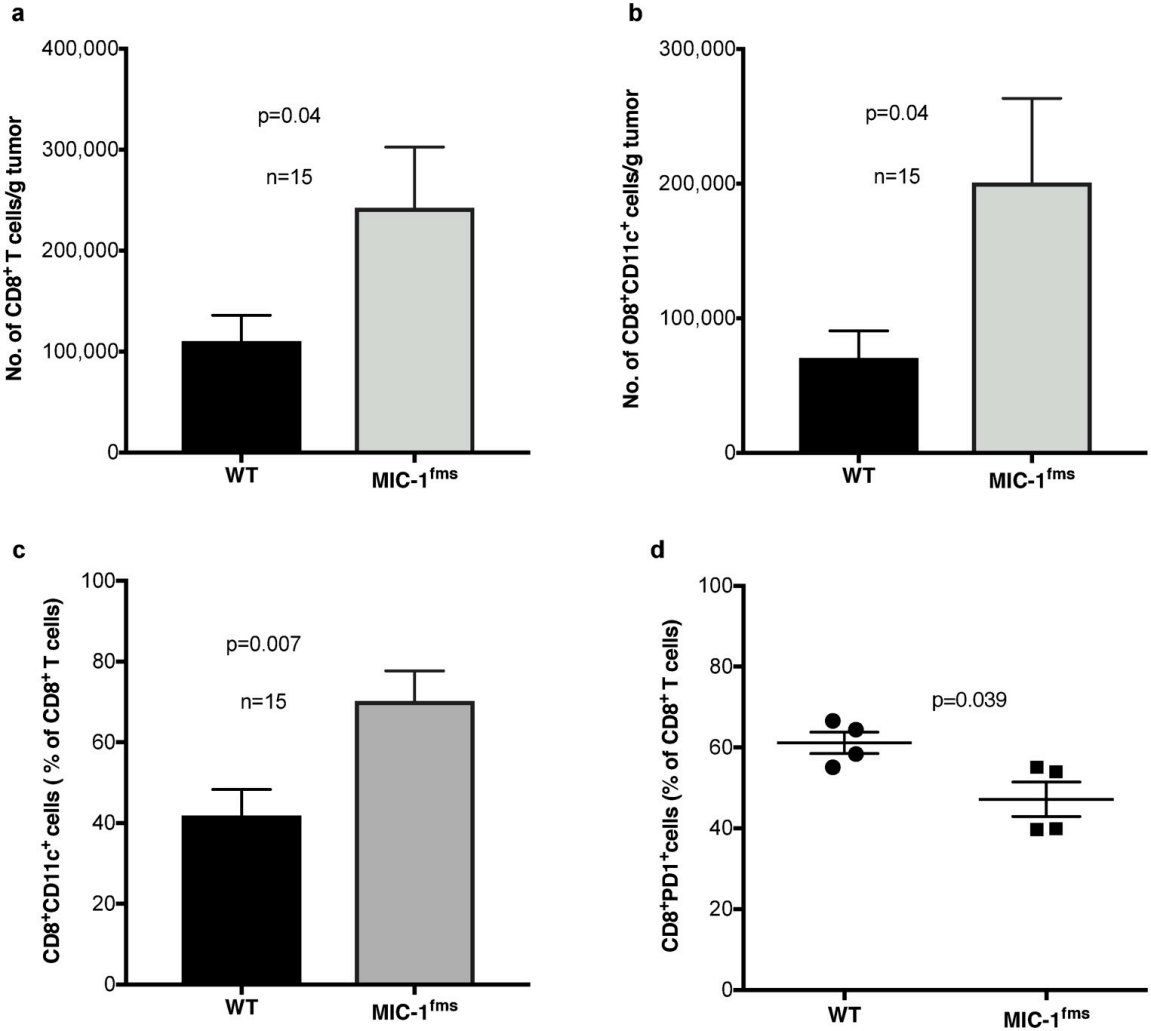
MIC^fms^ mice orthotopically transplanted with TRAMP tumors display altered CD8 T cell subsets. TRAMP^MIC-/-^ primary prostate tumor cells were engrafted orthotopically into WT and MIC-1^fms^ mice and 35 days later mice were sacrificed and cells from a known proportion of the orthotopically engrafted prostate tumor was subjected to multiparameter flow cytometry. (a) Number of tumor infiltrating CD3^+^CD8^+^T cells/g of intraprostatic tumor from WT and MIC-1^fms^ mice. (b) Number of tumor infiltrating CD3^+^CD8^+^CD11c^+^ T cells/g of intraprostatic tumor from WT and MIC-1^fms^ mice. (c) Proportion of tumor infiltrating CD3^+^CD8^+^ T cells also expressing CD11c in intraprostatic tumors from WT and MIC-1^fms^ mice. (d) Proportion of tumor infiltrating CD3^+^CD8^+^ T cells expressing PD1 in the intraprostatic tumor from WT and MIC-1^fms^ mice. All data are presented as mean cell number or percentage ± s.e.m. Data was analyzed using unpaired test and *p* value are shown on the graphs.

The overall results, reported in Table 1, indicate that the MIC-1^fms^ tumors have a significantly higher proportion of T cells (48.3±0.28% versus 40.2±2.7%; *p* = 0.048) and more than twice the number of T cells/g of tumor compared to WT tumors (7.30x10^5^±1.9x10^5^ versus 3.18x10^5^±0.7x10^5^; *p* = 0.047). Tumors from MIC-1^fms^ mice also had almost 2 fold more CD8^+^ T cells/g tumor in comparison to tumors from WT mice (Table 1, Fig 3a; *p* = 0.044). There was a trend for an increase in the number of CD4 T cells/g of tumor, but this fell just short of statistical significance (Table 1, *p* = 0.07). The number of CD4^-^CD8^-^ T cells/g of tumor, that may represent gamma delta T cells were more than 2 fold higher in MIC-1^fms^ tumors than WT tumors. There was no difference in the absolute number of B or NK cells but MIC-1^fms^ tumors have significantly higher proportion of NK cells than WT tumors (Table 1, *p* = 0.043).

**Table 1:** Number (per gram tumor) and proportion of tumor infiltrating lymphocytes in intraprostatic tumors from WT and MIC-1^fms^ mice. TRAMP^MIC-/-^ primary prostate tumor cells were engrafted orthotopically into WT and MIC-1^fms^ mice and 35 days later mice were sacrificed and cells from a known proportion of the orthotopically engrafted prostate tumor was subjected to multiparameter flow cytometry. Numbers and proportion of cells between two groups were compared using unpaired *t* tests (GraphPad Prism software version 7,GraphPad Software, San Diego, CA, USA). All data are presented as mean cell number or percentage ± s.e.m.

	Number of cells/g tumor ± s.e.m. x 10^3^			Lymphocytes	% Cells ± s.e.m.		
	WT Tumor	MIC-1^fms^ Tumor	p value		WT Tumor	MIC-1^fms^ Tumor	p value
**T cells**							
CD3^+^	318 ± 68	730 ± 191	0.047	CD3^+^ (% of CD45^+^ cells)	40.2 ± 2.7	48.3 ± 2.8	0.048
CD4^+^	39 ± 10	99 ± 31	0.070	CD4^+^ (% of CD3T cells)	11.2 ± 0.9	13.1 ± 1.0	0.174
CD8^+^	110 ± 25	242 ±60	0.044	CD8^+^ (% of CD3T cells)	39.9 ± 3.5	45.02 ± 3.1	0.287
CD4^+^CD8^+^	5 ± 2	8 ± 3	0.415	CD4^+^CD8^+^ (% of CD3T cells)	1.3 ± 0.4	1.2 ± 0.2	0.949
CD4^-^CD8^-^	148 ± 29	329 ± 80	0.038	CD4^-^CD8^-^ (% of CD3T cells)	47.3 ± 3.6	45.5 ± 4.1	0.741
**B cells**							
B220^+^	61 ± 15	83 ± 16	0.299	B220^+^ (% of CD45^+^ cells)	8.2 ± 0.7	7.4 ± 0.4	0.319
**NK cells**							
NK1.1^+^	9 ± 2	29 ± 12	0.105	NK1.1^+^ (% of CD45^+^ cells)	0.8 ± 0.1	1.1 ± 0.3	0.043

### MIC-1^fms^ mice with orthotopically transplanted TRAMP tumors display altered CD8 T cell subsets suggestive of reduced "exhaustion"

CD8 T cell mediated tumor killing is an important mechanism of antitumor immunity. We have therefore examined in more detail the impact of GDF15 on CD8 T cells subpopulations in WT and MIC-1^fms^ mice implanted intraprostatically with TRAMP tumors. Surface expression of CD11c on CD8 T cells identifies a population of antigen stimulated cytotoxic effector cells that secrete large amounts of IFNg [39]. As well as having more CD8 T cells (Fig 3a), MIC-1^fms^ tumors have higher numbers of CD8^+^CD11c^+^ T cells/g tumor (Fig 3b, *p* = 0.04) and the proportion of these cells is also increased from 41.8±6.5% to 70.3±7.4% (Fig 3c, *p* = 0.007) of CD8 T cells.

Cells surface expression of the immune checkpoint protein PD1 is a hallmark of "exhausted" CD8 T cells that have a depressed capacity to mediate antitumor immunity. The CD8 T cells from MIC-1^fms^ mice showed a substantially reduced proportion of exhausted CD8^+^PD1^+^ T cells (Fig 3d, *p* = 0.039). Overall, these phenotypic changes in CD8 T cells from tumor bearing MIC-1^fms^ mice suggest that they are less exhausted and may have a greater capacity for tumor cell killing.

## Discussion

Using a robust transgenic model our data clearly shows that the GDF15 mediated protection from growth of the PCa that develops spontaneously in C57/BL6 TRAMP mice, is dependent on intact adaptive immunity and in mice with adoptively transferred TRAMP tumors this response is at least in part mediated by CD8 bearing cells that are almost certainly T cells. The improved survival and reduced tumor size that is seen in GDF15 overexpressing TRAMP^fmsmic^ mice is completely abrogated in triple transgenic TRAMP^fmsmic/rag-/-^ mice, that also lack adaptive immunity because of deletion of the *Rag1* gene (Figs 1a and 1b). This data is consistent with other studies, which demonstrate that immune system manipulation does, as expected, impact the progressions to TRAMP tumors [40–43].

Whilst transgenic mice that spontaneously develop cancer, such as TRAMP mice, display a biology that more closely corresponds to the biology of the cancer under study, they also suffer from some important disadvantages. Foremost amongst these is the time required to develop double and triple transgenic mouse lines and the length of time required for tumor development which in TRAMP mice is approximately 6 months. To overcome some of these limitations and thus to be able to investigate the role of GDF15 more efficiently, we have established the OTTEM model in which prostate tumors derived from mice carrying the TRAMP transgene are engrafted orthotopically into the dorsal lobe of the prostates of mice of various genotypes. This strategy allows the engraftment of multiple mice from a tumor derived from a single donor mouse as well as the much more rapid evolution of tumor development (4–6 weeks versus 6 months), whilst still utilizing a primary spontaneous TRAMP tumor.

Using this OTTEM model, we can demonstrate similar GDF15 mediated, adaptive immunity dependent changes in tumor development, as that seen using spontaneous cancer development in TRAMP and TRAMP^fmsmic^ mice (Fig 1d). This GDF15 mediated protection from tumor growth is even more marked when the orthotopically engrafted cells come from TRAMP^rag-/-^ whose tumor has not undergone editing by the immune system prior to engraftment (Fig 1c). The effect of transgenic GDF15 overexpression can be reproduced by administering recombinant muGDF15 (Fig 1e) Further, consistent with GDF15 actions on adaptive immunity, the effect of recombinant muGDF15 infusion is additive to the protection offered by a monoclonal antibody to the immune checkpoint protein PD1 (Fig 2b). Thus, using multiple approaches our data indicates that the GDF15 protection from early TRAMP tumor growth requires adaptive immunity including CD8 T cells. Further, because the effects of GDF15 can be reproduced using systemically delivered GDF15 protein, it might be suitable as a therapeutic stimulator of antitumor immunity.

The direct target immune cell for the action of GDF15 is still uncertain. However, by direct or indirect mechanisms, its ultimate impact is likely to be at least in part on the CD8 T cells. Evidence for this is data that a monoclonal antibody to CD8, which causes CD8 T cell depletion, reverses the protective actions of GDF15 (Fig 2a). The phenotypic changes in CD8 T cells we have identified are also consistent with GDF15 stimulation of tumor immunity. The absolute number and proportion of tumor infiltrating CD3 expressing T cells is increased and also the absolute number of its CD8 subset, but the proportion of CD8 cells is not increased. Further, a more detailed evaluation of these CD8 T cells from tumors from GDF15 overexpressing mice indicates they have both a higher number and proportion of CD8^+^CD11c^+^ cells, a population that is thought to have been recently activated and produce large amounts of IFNg (Fig 3b and 3c). They also have more CD8 cells with reduced cell surface expression of PD1, a marker of T cell exhaustion (Fig 3d).

Overall these studies indicate that GDF15 overexpression or administration of recombinant muGDF15 leads to protection from the progress of spontaneous PCa in TRAMP and development of tumors in OTTEM mice that is dependent on having intact adaptive immunity and the presence of CD8 T cells. Additionally, muGDF15 infusion has at least an additive effect with checkpoint blocker therapy. This suggests that further studies may be warranted to determine if GDF15 has application as a stimulator of tumor immunity, in the therapy of cancer.

## Supporting information

S1 Data (PDF)

S1 Fig Gating strategy for the cells from prostate tumor. (PDF)

S2 Fig Gating strategy for the CD3^+^CD8^+^ T cells expressing PD1 in the prostate tumor. (PDF)

S1 Table List of antibodies used for flow cytometry. (DOCX)

S2 Table Effect of anti-CD8-alpha treatment of mice on CD8+ T cells proportions. (DOCX)
