# Supplementary material for: Growth differentiation factor-15 slows the growth of murine prostate cancer by stimulating tumor immunity
Source: PLoS One. 2020 Jun 5;15(6):e0233846. doi: 10.1371/journal.pone.0233846 (PMC7274405; doi:10.1371/journal.pone.0233846)
Supplement: S1 Data — (PDF) [file pone.0233846.s001.pdf]

Growth Differentiation Factor-15 slows the growth of murine prostate cancer by stimulating tumor immunity

Yasmin Husaini[1], Vicky Wang-Wei Tsai[1], Rakesh Manandhar[1], Hong Ping Zhang[1], Ka Ki Michelle Lee-Ng[1], Hélène Lebhar[2], Christopher P. Marquis[2], David A. Brown[1,3], Samuel N. Breit[1*].

Supplementary methods

Mouse genotyping

Transgenic mice were identified by PCR on DNA extracted from tail samples, using the transgene specific primers identified in the identified below.

1. For PB-SV40 T antigen sequence in TRAMP mice:

Pb-forward: 5'-CCGGTCGACCGGAAGCTTCCACAAGTGCATTTA-3' and

SV40Tag-reverse: 5'-CTCCTTTCAAGACCTAGAAGGTCCA-3'.

2. For GDF15 transgene in TRAMP$^{fmsmic-1}$ mice:

171MIC1 Flag-forward: 5'-GACTACAAGGACGACGATGACAAG-3' and

172MIC1MS8-reverse: 5'-CTCGGTGCACGCGGTAGGCTTCG-3'.

3. For RAG-KO mice:

1179_RAG1-KO-U1: TGGATGTGGAATGTGTGCGAG

1188_RAG1-WT-U1: GAGGTTCCGCTACGACTCTG

1189_RAG1-WT-L1: CCGGACAAGTTTTCATCGT

Detection of TRAMP tumor infiltrating lymphocytes

Lymphocytes were identified following the gating strategy shown in S1 Fig. After identifying leukocytes using anti-CD45$^+$, the antibodies used in Supplementary Table S1 identified T cells (CD3$^+$), CD4 T cells (CD3$^+$CD4$^+$) CD8 T cells (CD3$^+$CD8$^+$), CD11c expressing CD8 cells (CD3$^+$CD8$^+$CD11c$^+$), T cells bearing both CD4 and

CD8 ($CD3^+CD4^+CD8^+$), T cells with no CD4 or CD8 which may be gamma delta T cells ($CD3^+CD4^-CD8^-$), NK cells ($CD3^-CD11c^-CD11b^-B220^-NK1.1^+$) and B cells ($CD3^-CD11c^-CD11b^-B220^+NK1.1^-$).

***In vivo* CD8 T cell depletion**

To validate CD8 T depletion procedure, we depleted CD8 T cells from WT mice by twice weekly intraperitoneal injection of 400µg of rat anti-CD8-α monoclonal antibody (clone 2.43, Bio X Cell). A control was similarly injected with 400ug of isotype matched control rat anti KLH antibody (Clone LTF-2, Bio X Cell). After one week spleen and axillary and brachial lymph nodes were collected, dispersed to single cell suspension and 1 million cells were stained with CD45-PerCP, CD3-BV21 and CD8B-BV786 (Clone H35-17.2, BD Biosciences) and subjected to flow cytometry. Flow cytometry results indicated that there was essentially depletion of CD8 T cells in both spleen and lymph nodes (S2 Table).
