# Supplementary figures and images for: Growth differentiation factor-15 slows the growth of murine prostate cancer by stimulating tumor immunity
Source: PLoS One. 2020 Jun 5;15(6):e0233846. doi: 10.1371/journal.pone.0233846 (PMC7274405; doi:10.1371/journal.pone.0233846)

# Lymphocytes

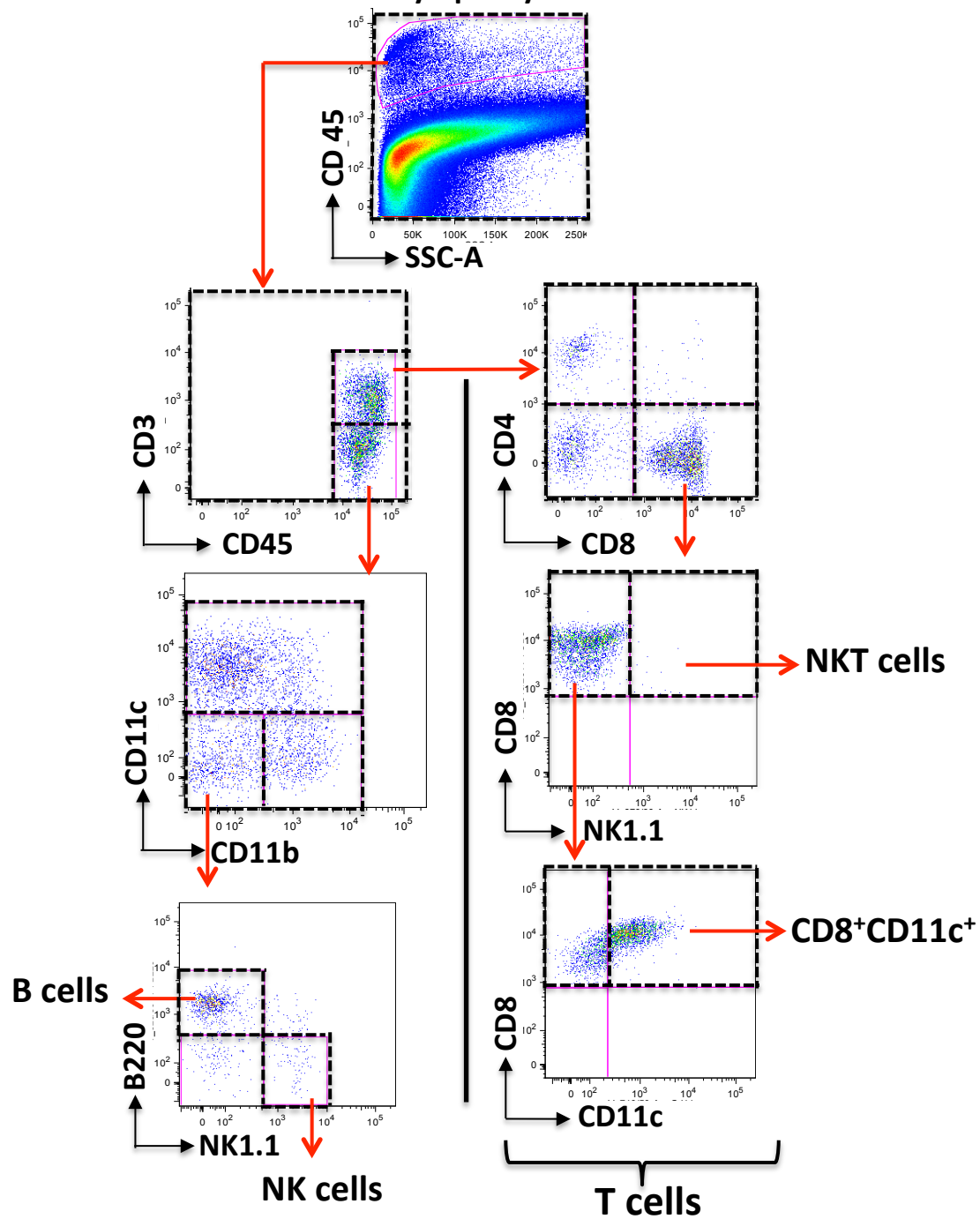

Supplement: S1 Fig — (PDF) [file pone.0233846.s002.pdf]

# Lymphocytes

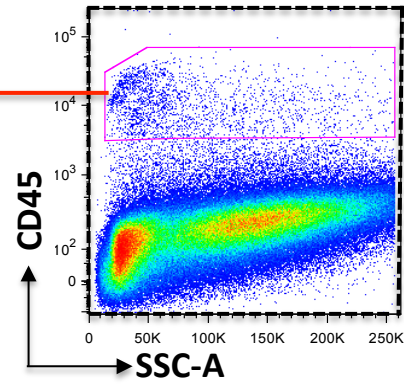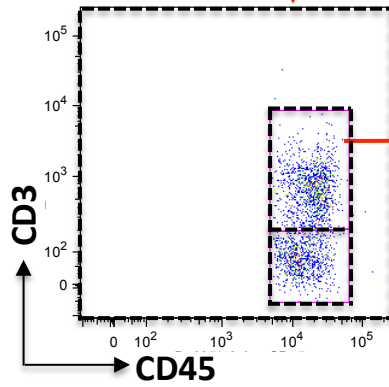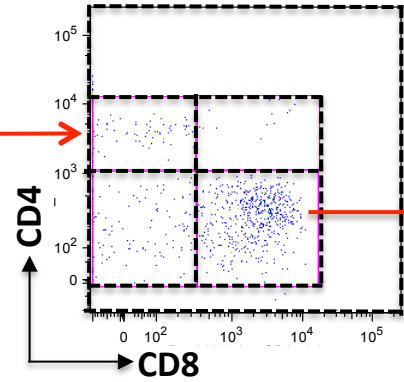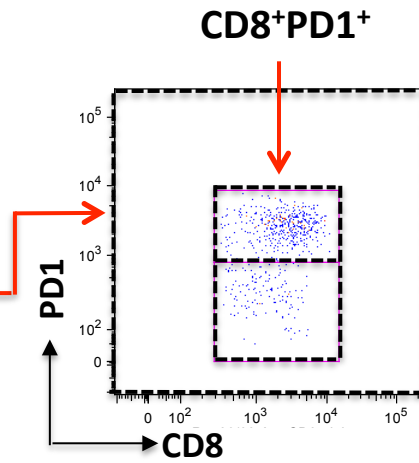

Supplement: S2 Fig — (PDF) [file pone.0233846.s003.pdf]
