# Supplementary material for: Growth differentiation factor-15 slows the growth of murine prostate cancer by stimulating tumor immunity
Source: PLoS One. 2020 Jun 5;15(6):e0233846. doi: 10.1371/journal.pone.0233846 (PMC7274405; doi:10.1371/journal.pone.0233846)
Supplement: S1 Table — (DOCX) [file pone.0233846.s004.docx]

| **S1 Table: List of antibodies used for flow cytometry** | | | |  |  |
| --- | --- | --- | --- | --- | --- |
|  |  |  |  |  |  |
| **Antibody** | **Conjugate** | **Clone** | **Cat No** | **RRID** | **Source** |
| 16/CD32 | Fc block | 2.4G2 | 553142 | AB_394657 | BD Biosciences |
| *Panel-1* |  |  |  |  |  |
| CD45 | FITC | 30-F11 | 553079 | AB_394609 | BD Biosciences |
| CD3e | BV421 | 145-2C11 | 562600 | AB_11153670 | BD Biosciences |
| CD4 | BV786 | RM4-5 | 563727 | AB_2728707 | BD Biosciences |
| CD8-α | PECF594 | 53-6.7 | 562315 | AB_11154579 | BD Biosciences |
| B220 | V500 | RA3-6B2 | 561227 | AB_10562193 | BD Biosciences |
| NK1.1 | BV650 | PK136 | 564143 |  | BD Biosciences |
| CD11c | BV711 | HL3 | 563048 |  | BD Biosciences |
| CD11b | APCCy7 | M1/70 | 557657 | AB_396772 | BD Biosciences |
| *Panel 2* |  |  |  |  |  |
| CD45 | PerCp-Cy5.5 | 30-F11 | 550994 | AB_394003 | BD Biosciences |
| CD3e | BV421 | 145-2C11 | 562600 | AB_11153670 | BD Biosciences |
| CD4 | BV786 | RM4-5 | 563727 | AB_2728707 | BD Biosciences |
| CD8-alpha | APCCy7 | 53-6.7 | 557654 | AB_396769 | BD Biosciences |
| CD279 (PD-1) | AF 647 | RMP1-30 | 109118 | AB_2566550 | BioLegend |
|  |  |  |  |  |  |
