# Supplementary material for: Growth differentiation factor-15 slows the growth of murine prostate cancer by stimulating tumor immunity
Source: PLoS One. 2020 Jun 5;15(6):e0233846. doi: 10.1371/journal.pone.0233846 (PMC7274405; doi:10.1371/journal.pone.0233846)
Supplement: S2 Table — (DOCX) [file pone.0233846.s005.docx]

| **S2 Table: Effect of anti-CD8-alpha treatment of mice on CD8^+^ T cells proportions.** | | | | | | | |
| --- | --- | --- | --- | --- | --- | --- | --- |
|  |  | |  | |  | |  |
|  | | **Isotype Control treated** | | | **Anti-CD8-alpha treated** | | |
|  | |  | |  |  |  | |
| **Tissue** | | **% CD3^+^ T cells** | | **CD8^+^ T cells as % of CD3** | **% CD3^+^ T cells** | **% CD8^+^ T cells as % of CD3** | |
|  | |  | |  |  |  | |
| Spleen | | 29.75±0.35 | | 37.7±0.21 | 21.75±0.04 | 0.05±0.07 | |
|  | |  | |  |  |  | |
| Lymph nodes | | 81.35±0.21 | | 44.35±0.21 | 74.3±0.42 | 0.00±0.00 | |
