# Supplementary material for: Growth differentiation factor-15 slows the growth of murine prostate cancer by stimulating tumor immunity
Source: PLoS One. 2020 Jun 5;15(6):e0233846. doi: 10.1371/journal.pone.0233846 (PMC7274405; doi:10.1371/journal.pone.0233846)
Supplement: Attachment — Submitted filename: Response to reviewers.docx [file pone.0233846.s006.docx]

**Response to Reviewer and Editors Comments**

1. There are numerous typographical errors in the manuscript.

We have corrected all typographical errors we have been able to identity

2. Authors should write appropriate scientific names of genes such "Tgfb  should be written as TGF beta (or symbol).

We have replaced TGF-b with TGF-beta.

3. Authors should write correctly the genes of mouse and human origin as per scientific nomenclature. In the manuscript there is no distinction between human and mouse genes and appears confusing in various places.

We have corrected the representation of genes in this manuscript using official designations. For example the gene for murine GDF15 becomes Gdf15. Additionally we have clarified the species of GDF15 protein used in the studies e.g. GDF15 becomes muGDF15.

1. As per our survival studies guidelines please amend the Methods section which describes humane endpoints by adding the number of mice that reached these endpoints or were found dead, if any.

We have amended the methods on P8, L164-166, as follows:

Out of 35 mice per group, 3 mice in TRAMP group, 4 mice in TRAMP^fmsmic-1^ group, 3 mice in TRAMP^rag-/-^ group and 5 mice in TRAMP^fmsmic/rag1-/-^ group were euthanized for the reasons other than tumor growth.

2. We note that you have a patent relating to material pertinent to this article. Please provide an amended statement of Competing Interests to declare this patent

Please note that the numbers of patents are too numerous to list individually as there are multiple patent families and each family has a different number in each different country/jurisdiction. Because of this, in our last PLOS ONE publication (Husaini, Y et al (2015) PLoS One **10**:e0115189.) we had agreed on the format below, which I hope you find acceptable.

DAB and SNB are named inventors on patents owned by St Vincent’s Hospital that pertain to the clinical use of a MIC-1/GDF15 diagnostic assay and modulatory therapy. DAB and SNB declare no competing non-financial interests. YH, VWWT, RM, HPZ, KMLN, HL and CPM declare no competing financial and/or nonfinancial interests. St Vincent’s Hospital agrees to make freely available any materials and information described in this publication that may be reasonably requested for the purpose of academic, non-commercial research. Due to the proprietary nature of the materials, the parties will need to enter into a material transfer agreement. This does not alter the authors’ adherence to all the PLOS ONE policies on sharing data and materials.

3. PLOS requires an ORCID iD for the corresponding author in Editorial Manager on papers submitted after December 6th, 2016

Samuel Breit's ORCID ID is 0000-0002-9021-9879
