# Supplementary material for: Growth differentiation factor-15 slows the growth of murine prostate cancer by stimulating tumor immunity
Source: PLoS One. 2020 Jun 5;15(6):e0233846. doi: 10.1371/journal.pone.0233846 (PMC7274405; doi:10.1371/journal.pone.0233846)
Supplement: Peer review history

# Peer review: Decision Letter 0

**DOI:** 10.1371/journal.pone.0233846.r001

**Authors:** MOHAMMAD Saleem

1 Apr 2020

PONE-D-19-32258

Growth Differentiation Factor-15 slows the growth of murine prostate cancer by stimulating tumor immunity

PLOS ONE

Dear Dr Breit:

Thank you for submitting your manuscript to PLOS ONE. After careful consideration, we feel that it has merit but does not fully meet PLOS ONE’s publication criteria as it currently stands. Therefore, we invite you to submit a revised version of the manuscript that addresses the points raised during the review process. During the review process, we identified this manuscript very informative and identified some minor issues which need to be addressed. These are as follows:

1. There are numerous typographical errors in the manuscript.

2. Authors should write appropriate scientific names of genes such "Tgfb  should be written as TGF beta (or symbol).

3. Authors should write correctly the genes of mouse and human origin as per scientific nomenclature. In the manuscript there is no distinction between human and mouse genes and  appears confusing in various places.

Please include the following items when submitting your revised manuscript:

- A rebuttal letter that responds to each point raised by the academic editor and reviewer(s). This letter should be uploaded as separate file and labeled 'Response to Reviewers'.
- A marked-up copy of your manuscript that highlights changes made to the original version. This file should be uploaded as separate file and labeled 'Revised Manuscript with Track Changes'.
- An unmarked version of your revised paper without tracked changes. This file should be uploaded as separate file and labeled 'Manuscript'.

Please note while forming your response, if your article is accepted, you may have the opportunity to make the peer review history publicly available. The record will include editor decision letters (with reviews) and your responses to reviewer comments. If eligible, we will contact you to opt in or out. We look forward to receiving your revised manuscript.

Kind regards,

M. Saleem

Academic Editor

PLOS ONE

Journal requirements:

When submitting your revision, we need you to address these additional requirements.

Please ensure that your manuscript meets PLOS ONE's style requirements, including those for file naming. The PLOS ONE style templates can be found at

https://journals.plos.org/plosone/s/file?id=wjVg/PLOSOne_formatting_sample_main_body.pdf and

https://journals.plos.org/plosone/s/file?id=ba62/PLOSOne_formatting_sample_title_authors_affiliations.pdf

1. As per our survival studies guidelines please amend the Methods section which describes humane endpoints by adding the number of mice that reached these endpoints or were found dead, if any.

2. We note that you have a patent relating to material pertinent to this article. Please provide an amended statement of Competing Interests to declare this patent (with details including name and number), along with any other relevant declarations relating to employment, consultancy, patents, products in development or modified products etc. Please confirm that this does not alter your adherence to all PLOS ONE policies on sharing data and materials, as detailed online in our guide for authors http://journals.plos.org/plosone/s/competing-interests by including the following statement: "This does not alter our adherence to  PLOS ONE policies on sharing data and materials.” If there are restrictions on sharing of data and/or materials, please state these. Please note that we cannot proceed with consideration of your article until this information has been declared.

This information should be included in your cover letter; we will change the online submission form on your behalf.

Please know it is PLOS ONE policy for corresponding authors to declare, on behalf of all authors, all potential competing interests for the purposes of transparency. PLOS defines a competing interest as anything that interferes with, or could reasonably be perceived as interfering with, the full and objective presentation, peer review, editorial decision-making, or publication of research or non-research articles submitted to one of the journals. Competing interests can be financial or non-financial, professional, or personal. Competing interests can arise in relationship to an organization or another person. Please follow this link to our website for more details on competing interests: http://journals.plos.org/plosone/s/competing-interests

3. PLOS requires an ORCID iD for the corresponding author in Editorial Manager on papers submitted after December 6th, 2016. Please ensure that you have an ORCID iD and that it is validated in Editorial Manager. To do this, go to ‘Update my Information’ (in the upper left-hand corner of the main menu), and click on the Fetch/Validate link next to the ORCID field. This will take you to the ORCID site and allow you to create a new iD or authenticate a pre-existing iD in Editorial Manager. Please see the following video for instructions on linking an ORCID iD to your Editorial Manager account: https://www.youtube.com/watch?v=_xcclfuvtxQ

# Author comment: Author response to Decision Letter 0

**DOI:** 10.1371/journal.pone.0233846.r002

12 May 2020

Response to Reviewer and Editors Comments

1. There are numerous typographical errors in the manuscript. 

Response: We have corrected all typographical errors we have been able to identity

2. Authors should write appropriate scientific names of genes such "Tgfb should be written as TGF beta (or symbol).

Response: We have replaced TGF-b with TGF-beta.

3. Authors should write correctly the genes of mouse and human origin as per scientific nomenclature. In the manuscript there is no distinction between human and mouse genes and appears confusing in various places.

Response: We have corrected the representation of genes in this manuscript using official designations. For example the gene for murine GDF15 becomes Gdf15. Additionally we have clarified the species of GDF15 protein used in the studies e.g. GDF15 becomes muGDF15.

1. As per our survival studies guidelines please amend the Methods section which describes humane endpoints by adding the number of mice that reached these endpoints or were found dead, if any.

Response:We have amended the methods on P8, L164-166, as follows:

Out of 35 mice per group, 3 mice in TRAMP group, 4 mice in TRAMPfmsmic-1 group, 3 mice in TRAMPrag-/- group and 5 mice in TRAMPfmsmic/rag1-/- group were euthanized for the reasons other than tumor growth.

2. We note that you have a patent relating to material pertinent to this article. Please provide an amended statement of Competing Interests to declare this patent

Response:Please note that the numbers of patents are too numerous to list individually as there are multiple patent families and each family has a different number in each different country/jurisdiction. Because of this, in our last PLOS ONE publication (Husaini, Y et al (2015) PLoS One 10:e0115189.) we had agreed on the format below, which I hope you find acceptable.

DAB and SNB are named inventors on patents owned by St Vincent’s Hospital that pertain to the clinical use of a MIC-1/GDF15 diagnostic assay and modulatory therapy. DAB and SNB declare no competing non-financial interests. YH, VWWT, RM, HPZ, KMLN, HL and CPM declare no competing financial and/or nonfinancial interests. St Vincent’s Hospital agrees to make freely available any materials and information described in this publication that may be reasonably requested for the purpose of academic, non-commercial research. Due to the proprietary nature of the materials, the parties will need to enter into a material transfer agreement. This does not alter the authors’ adherence to all the PLOS ONE policies on sharing data and materials.

3.PLOS requires an ORCID iD for the corresponding author in Editorial Manager on papers submitted after December 6th, 2016

Samuel Breit’s ORCID ID is 0000-0002-9021-9879

Attachment Submitted filename: Response to reviewers.docx Click here for additional data file.

# Editor report: Decision Letter 1

**DOI:** 10.1371/journal.pone.0233846.r003

**Authors:** MOHAMMAD Saleem

14 May 2020

Growth Differentiation Factor-15 slows the growth of murine prostate cancer by stimulating tumor immunity

PONE-D-19-32258R1

Dear Dr. Samuel N Breit,

We are pleased to inform you that your manuscript has been judged scientifically suitable for publication and will be formally accepted for publication once it complies with all outstanding technical requirements.

Within one week, you will receive an e-mail containing information on the amendments required prior to publication. When all required modifications have been addressed, you will receive a formal acceptance letter and your manuscript will proceed to our production department and be scheduled for publication.

Shortly after the formal acceptance letter is sent, an invoice for payment will follow. To ensure an efficient production and billing process, please log into Editorial Manager at https://www.editorialmanager.com/pone/, click the "Update My Information" link at the top of the page, and update your user information. If you have any billing related questions, please contact our Author Billing department directly at authorbilling@plos.org.

If your institution or institutions have a press office, please notify them about your upcoming paper to enable them to help maximize its impact. If they will be preparing press materials for this manuscript, you must inform our press team as soon as possible and no later than 48 hours after receiving the formal acceptance. Your manuscript will remain under strict press embargo until 2 pm Eastern Time on the date of publication. For more information, please contact onepress@plos.org.

With kind regards,

Mohammad Saleem 

Academic Editor, PLOS ONE

Masonic Cancer Center, University of Minnesota, Minneapolis, USA

# Editor report: Acceptance letter

**DOI:** 10.1371/journal.pone.0233846.r004

**Authors:** MOHAMMAD Saleem

28 May 2020

PONE-D-19-32258R1 

Growth Differentiation Factor-15 slows the growth of murine prostate cancer by stimulating tumor immunity 

Dear Dr. Breit:

I am pleased to inform you that your manuscript has been deemed suitable for publication in PLOS ONE. Congratulations! Your manuscript is now with our production department. 

If your institution or institutions have a press office, please notify them about your upcoming paper at this point, to enable them to help maximize its impact. If they will be preparing press materials for this manuscript, please inform our press team within the next 48 hours. Your manuscript will remain under strict press embargo until 2 pm Eastern Time on the date of publication. For more information please contact onepress@plos.org.

For any other questions or concerns, please email plosone@plos.org. 

Thank you for submitting your work to PLOS ONE.

With kind regards,

PLOS ONE Editorial Office Staff

on behalf of

Dr. MOHAMMAD Saleem 

Academic Editor

PLOS ONE
